# Usefulness of artificial vascular graft for venous reconstruction in liver surgery

**DOI:** 10.1186/1477-7819-12-113

**Published:** 2014-04-23

**Authors:** Tatsuya Orimo, Toshiya Kamiyama, Hideki Yokoo, Tatsuhiko Kakisaka, Kenji Wakayama, Yosuke Tsuruga, Hirofumi Kamachi, Akinobu Taketomi

**Affiliations:** 1 Department of Gastroenterological Surgery I, Hokkaido University Graduate School of Medicine, North 15-West 7, Kita-Ku, 060-8638 Sapporo, Hokkaido, Japan

**Keywords:** Artificial vascular graft, ePTFE graft, Hepatectomy, Liver surgery

## Abstract

**Background:**

The purpose of this study was to evaluate the results of hepatectomy with inferior vena cava or hepatic vein resection, followed by vessel reconstruction with an artificial vascular graft.

**Methods:**

From 2000 to 2011, 1,434 patients underwent several types of hepatectomy at our institution. Of these, we reviewed the cases of eight patients (0.56%) who underwent hepatectomy with inferior vena cava or hepatic vein resection and subsequent reconstruction using an expanded polytetrafluoroethylene (PTFE) graft.

**Results:**

We resected the inferior vena cava in six patients and the hepatic vein in two patients. All eight patients underwent subsequent reconstruction using an expanded PTFE graft. The median operative time was 443 minutes and the median blood loss was 2,017 mL. The median postoperative hospital stay period was 18.5 days and the in-hospital mortality rate was 0%. Complications occurred in four patients: two patients experienced bile leakage, one experienced a wound infection, and one experienced pleural effusion. The two patients who experienced bile leakage had undergone reoperation on postoperative day 1. No complication with the artificial vascular graft occurred in these eight cases. Histological invasion to the replaced inferior vena cava or hepatic vein was confirmed in four cases. All artificial vascular grafts remained patent during the observation period.

**Conclusions:**

Hepatectomy combined with inferior vena cava or hepatic vein resection, followed by reconstruction with an expanded PTFE graft can be performed safely in selected patients.

## Background

Liver resection is often the only valid treatment for patients with hepatic tumors such as hepatocellular carcinomas, cholangiocarcinomas and metastatic hepatic carcinomas. Whether hepatectomy can be performed often influences the prognosis of these patients. If the liver tumor is otherwise unresectable, liver surgery requiring venous resection and reconstruction may be the only chance for a cure. Liver tumors invading the inferior vena cava (IVC) or hepatic vein (HV) roots were previously a contraindication for hepatectomy because of the high surgical risk, but along with the progress in liver transplantation, complex hepatectomies, such as those with vein reconstruction, have also become relatively safe [1]. When a liver tumor invades the IVC or HV, a liver resection combined with simple closure or patch reconstruction of the IVC or HV is performed when the range of infiltration is small. However, when the range of the infiltration is large, a graft is required. HV reconstruction is also occasionally required to reduce the residual liver congestion area and ensure an effective residual liver volume. However, in terms of liver surgery using an artificial vascular graft, there are few reports and many uncertainties about the results, prognosis and graft patency.

In this study, we reviewed the cases of eight patients who underwent hepatectomy with IVC or HV resection and subsequent reconstruction using an expanded polytetrafluoroethylene (ePTFE) graft between 2000 and 2011. The results validate the use of the artificial vascular graft in liver surgery.

## Methods

### Patients

From 2000 to 2011, 1,434 patients underwent several types of hepatectomy at the Department of Gastroenterological Surgery I, Hokkaido University Hospital. Eight of these patients (0.56%) underwent hepatectomy with IVC or HV resection and subsequent reconstruction using an ePTFE graft (Gore-Tex, WL Gore & Associates, Inc., USA). Six of these patients needed IVC resection and two needed HV resection. The clinical and surgical features of the patients are listed in Table 1. There were six men and two women; their ages ranged from 50 to 84 years with a median age of 64.5 years. Three of the liver tumors were intrahepatic cholangiocarcinomas, two were hepatocellular carcinomas, and the remainder were single cases of metastatic tumor from colon cancer, metastatic tumor from gastrointestinal stromal tumor, and sarcomatoid mesothelioma. Patient 3 had a recurrent gastrointestinal stromal tumor in the residual liver (S4); this patient underwent a fourth hepatectomy, after a right hepatectomy and two partial resections of the liver. The other patients were primary surgical cases. The preoperative diagnosis of patient 5 was retroperitoneal leiomyosarcoma, but the postoperative histological diagnosis was sarcomatoid mesothelioma. Biliary reconstruction by Roux-en-Y hepaticojejunostomy was performed in a single case (patient 1). Informed consent was obtained from each patient in accordance with the Ethics Committee Guidelines at our institution.

**Table 1:** The clinical and surgical features of the patients in this study

**Patient number**	**Sex/age**	**Disease**	**Liver resection (resected segments)**	**Operation time (min)**	**Blood loss** **(mL)**	**Vascular resection**	**Other combined resection**	**Vascular graft**	**ICG R15 (%)**	**Oncological stage**
1	F/56	Intrahepatic cholangiocarcinoma	Right trisectionectomy (1, 4 to 8)	548	2285	IVC	Bile duct	20 mm ringed ePTFE tube graft	9.7	3
2	M/67	Intrahepatic cholangiocarcinoma	Left hepatectomy (1 to 4)	503	2230	IVC	None	20 mm ringed ePTFE tube graft	5	4A
3	F/68	Metastatic gastrointestinal stromal tumor	Partial resection (4)	331	820	IVC	None	20 mm ringed ePTFE tube graft	21.2	4
4	M/73	Hepatocellular carcinoma	Right trisectionectomy (1, 4 to 8)	419	3600	LHV	None	10 mm ringed ePTFE tube graft	15.2	2
5	M/50	Sarcomatoid mesothelioma	Right hepatectomy (1,5 to 8)	519	3600	IVC	Diaphragm, lower lobe of right lung	20 mm ringed ePTFE tube graft	3.3	4
6	M/53	Hepatocellular carcinoma	Left hepatectomy (1 to 4)	366	730	MHV	None	10 mm ringed ePTFE tube graft	13.2	3
7	M/84	Metastatic colon cancer	Partial resection (7)	414	1804	IVC	None	ePTFE patch graft	15.2	4
8	M/62	Intrahepatic cholangiocarcinoma	Partial resection (1)	467	1760	IVC	None	20 mm ringed ePTFE tube graft	13.4	3

ePTFE, expanded polytetrafluoroethylene; IVC, inferior vena cava; LHV, left hepatic vein; MHV, middle hepatic vein; ICG R15, indocyanine green retention rate at 15 min.

### Preoperative management

**Figure 1:**
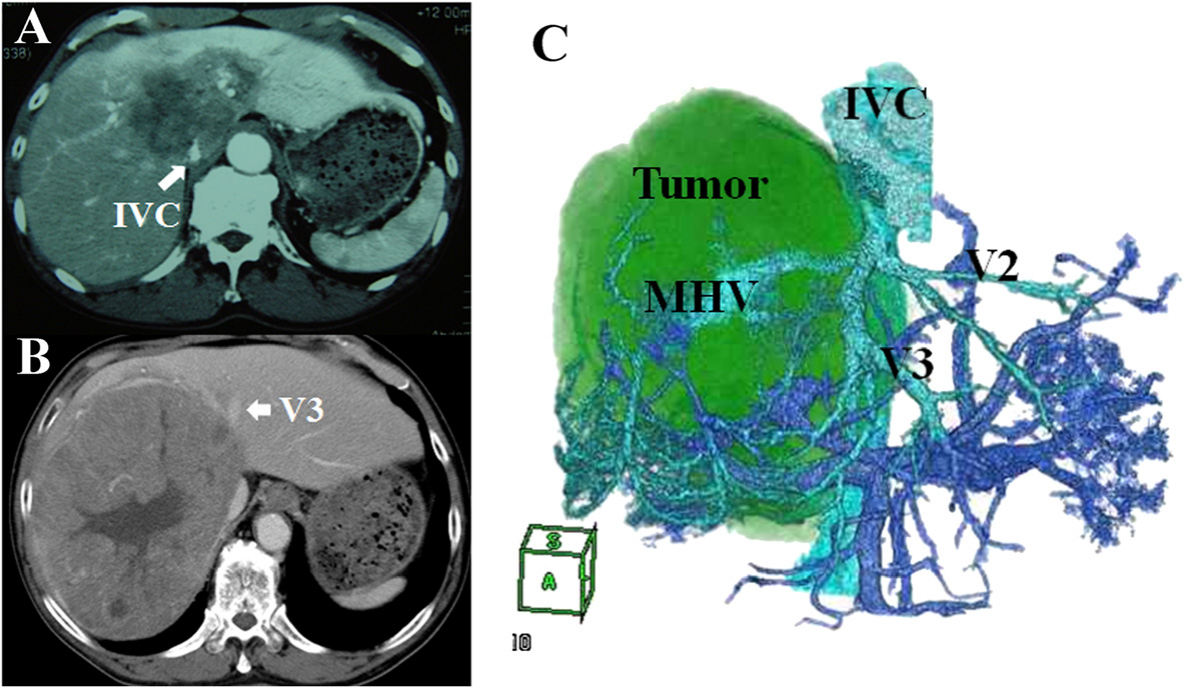
**Preoperative enhanced computed tomography and reconstructed three-dimensional computed tomography. (A)** Intrahepatic cholangiocarcinoma: tumor invasion of the inferior vena cava was suspected (patient 2). **(B)** Hepatocellular carcinoma: tumor invasion of the vein (V3) was suspected (patient 4). **(C)** Hepatocellular carcinoma: the left hepatic vein diverged to confluent V2 and V3 near the root and V3 was compressed by the tumor over a 3 cm length (patient 4). IVC, inferior vena cava; MHV, middle hepatic vein.

Preoperative management was done according to our previous report [2]. All patients were evaluated by abdominal and chest computed tomography (CT) to assess the primary tumor, vascular structures and distant metastases. In addition, we reconstructed three-dimensional (3D) images using multidetector row-CT to understand the positional relationships between the tumor and vessels in greater detail (Figure 1). The volumes of the liver parenchyma and tumors were measured using a 3D workstation and the effective resection ratio (%) was calculated. The indocyanine green retention rate at 15 min (ICGR15) was measured to evaluate the liver functional reserve. An algorithm (Hokkaido University Algorithm) incorporating the ICGR15 and remnant liver volume was used to determine the operative procedure, as previously described [2].

### Surgical methods

The surgical method used for the liver resections has been previously described [2]. Transection of the liver parenchyma was performed using the hook spatula of an ultrasonic harmonic scalpel (Ethicon EndoSurgery, San Angelo, Texas, USA) and either a DS3.0 Dissecting Sealer (Medtronic) or bipolar cautery with a saline irrigation system. Inflow occlusion was applied in an intermittent manner, with 15 min of occlusion alternated with 5 min of reperfusion. During transection of the liver parenchyma, the central venous pressure was maintained below 5 cm H_2_O to prevent venous hemorrhage. Liver resection in this study included two right trisectionectomies, two left hepatectomies, one right hepatectomy, and three partial resections.

**Figure 2:**
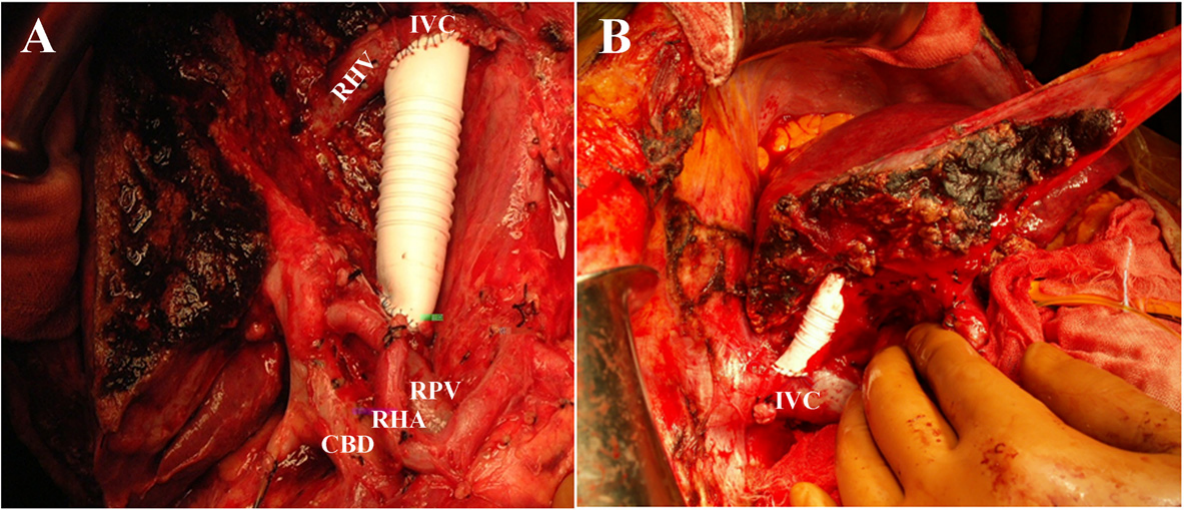
**Hepatectomy combined with inferior vena cava or hepatic vein resection followed by reconstruction with an artificial vascular graft. (A)** Left hepatectomy with reconstruction of the inferior vena cava using an expanded polytetrafluoroethylene (ePTFE, Gore-Tex, WL Gore&Associates, Inc., USA) graft (patient 2). **(B)** Right trisectionectomy with reconstruction of the left hepatic vein using an ePTFE graft (patient 4). CBD, common bile duct; IVC, inferior vena cava; RHA, right hepatic artery; RHV, right hepatic vein; RPV, right portal vein.

Starting with Kocher’s maneuver, the IVC was separated from the retroperitoneum and secured above the level of the right renal vein. After mobilizing the right and left liver lobes, the right and left sidewalls of the IVC were exposed, and the IVC was separated from the retroperitoneum and secured above the level of the HV roots. When IVC resection was necessary because of tumor invasion and a wall resection of the IVC involved less than a quarter of the entire circumference, the IVC was reconstructed by primary repair. When the wall resection of the IVC involved less than one-half of the entire circumference, the IVC was reconstructed with an umbilical vein patch graft or an ePTFE patch graft (*n* = 1). When the defect of the IVC wall was greater than one-half of the entire circumference, the IVC was reconstructed with a 20 mm ringed ePTFE tube graft (*n* = 5; Figure 2). When HV resection and reconstruction was necessary because the tumor invaded the HV and the planned residual liver volume was expected to be smaller than 40% of the whole liver, the HV was reconstructed with a 10 mm ringed ePTFE tube graft (*n* = 2; Figure 2). Resection and reconstruction of the IVC or HV were performed after transection of the liver parenchyma.

In reconstructing the IVC with an ePTFE graft, the IVC was clamped at both the suprahepatic and suprarenal portions. All the IVC segments replaced in these cases were retrohepatic IVC segments. During HV reconstruction, the ePTFE graft was bypassed either from V3 to the IVC (patient 4) or from the middle hepatic vein (MHV) to the IVC (patient 6). In reconstructing the IVC with an ePTFE graft, the IVC was clamped as follows: the IVC was clamped at both the suprarenal and suprahepatic regions, the liver and invaded IVC was removed, and the IVC was reconstructed with an ePTFE graft. After reconstruction, the IVC clamp was first released at the suprarenal region and later at the suprahepatic region for the air flush. In reconstructing the HV with an ePTFE graft, the IVC was clamped as follows: the IVC was clamped at both the suprarenal and suprahepatic regions and inflow occlusion was applied, and the HV was reconstructed with an ePTFE graft. After HV was reconstructed and inflow occlusion was released, the IVC clamp was first released at the suprarenal region and later at the suprahepatic region for the air flush. At our institution, venovenous bypass is used in case of a decrease in systolic blood pressure of less than 60 mmHg by test clamping the IVC even after volume loading. A venovenous bypass was not used for any the patients in this study because no cases had a decrease in blood pressure when clamping the IVC, although we were prepared to use the bypass.

### Postoperative management

No anticoagulant therapy was used for patients undergoing reconstruction of the IVC with an ePTFE graft. Anticoagulant therapy with warfarin was used to maintain an international normalized ratio of 1.5 to 2.0 for patients undergoing reconstruction of the HV with an ePTFE graft. Ultrasound sonography was performed daily for one week after surgery to check the graft patency. CT was performed on postoperative day 7 to evaluate the graft patency, and follow-up studies after discharge were conducted one month after the operation and at three-month intervals thereafter.

## Results

**Figure 3:**
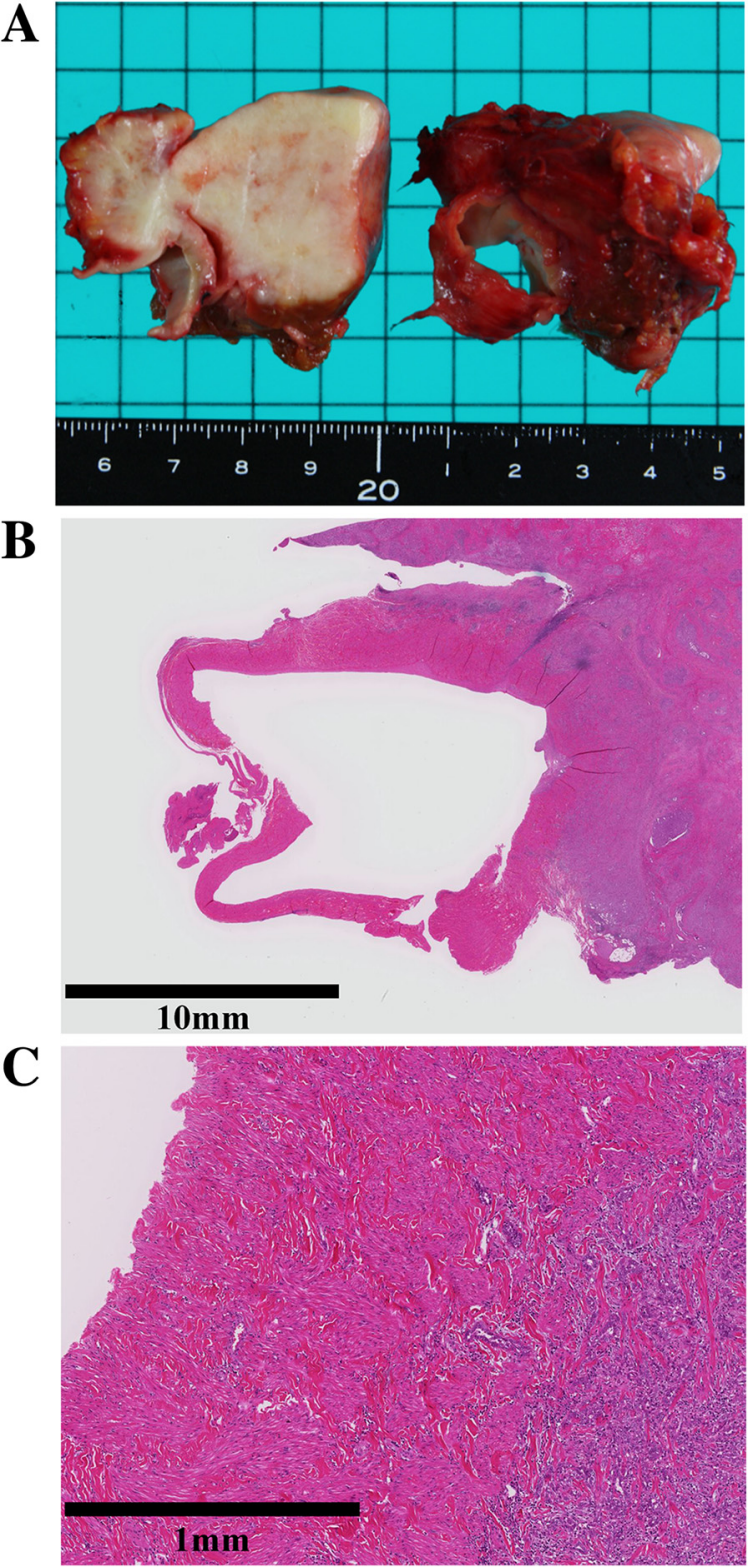
**Histological invasion to the replaced inferior vena cava by the tumor was confirmed (patient 8). (A)** Gross appearance **(B)** Low magnification. **(C)** High magnification.

**Figure 4:**
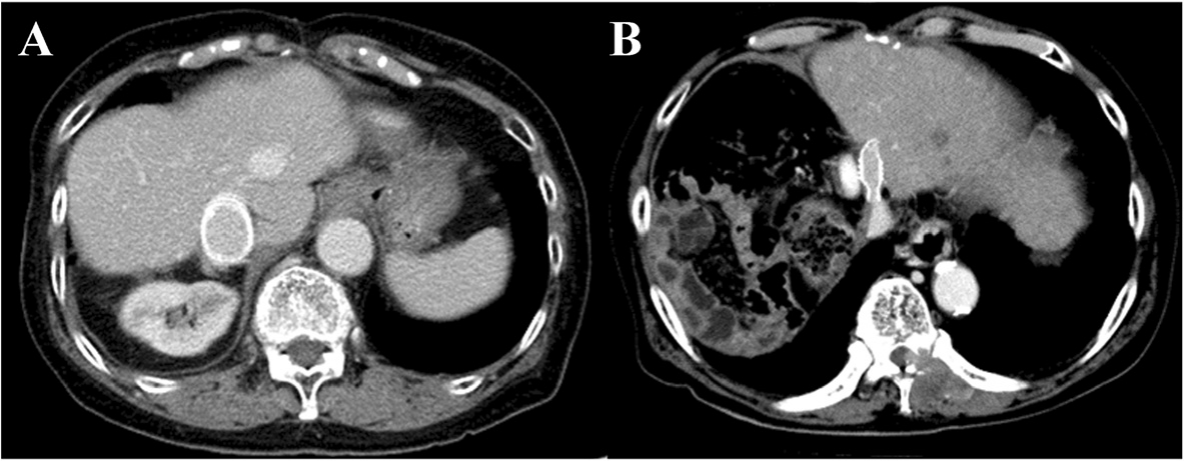
**Postoperative enhanced computed tomography. (A)** The patency of an inferior vena cava graft has been kept for nine years after the operation (patient 3). **(B)** The patency of a hepatic vein graft has been kept for one year after the operation (patient 4).

The details of the surgical results are listed in Table 2. We resected the IVC in six patients and the HV in two. All eight patients underwent subsequent reconstruction using an ePTFE graft. The median operative time was 443 minutes and the median blood loss was 2,017 mL. Other combined resected organs were the bile duct in one case and the diaphragm and right lung in another. Complications occurred in four patients: two patients experienced bile leakage, one experienced a wound infection, and one experienced pleural effusion. Patients 2 and 8 underwent reoperations to treat bile leakage on postoperative day 1. The median postoperative hospital stay period was 18.5 days and the in-hospital mortality rate was 0%. No complications with regard to the artificial vascular graft occurred in these patients. Histological invasion of the replaced IVC or HV was confirmed in four cases (patients 1, 5, 7 and 8; Figure 3). The median duration of follow up was 24 (range, 8 to 117) months. After surgery, CT or magnetic resonance imaging was performed at one- to three-month intervals to determine recurrence and check the graft patency. When the graft patency period was defined as the period from the operation to the last evidence of radiological patency, all artificial vascular grafts remained patent during the observation period (Figure 4).

**Table 2:** Surgical results of the patients in this study

**Patient number**	**Postoperative hospital stay (days)**	**Morbidity**	**Graft patency (period)**	**Outcome**	**Histological invasion to the replaced IVC or HV**
1	19	None	Patent (5y, 6mo)	Disease death (7y, 1mo)	Positive
2	8	Bile leakage	Patent (1y, 3mo)	Disease death (1y, 3mo)	Negative
3	7	None	Patent (9y, 9mo)	Alive (9y, 9mo)	Negative
4	19	Wound infection	Patent (1y, 5mo)	Disease death (2y)	Negative
5	19	Pleural effusion	Patent (5mo)	Disease death (8mo)	Positive
6	16	None	Patent (7mo)	Disease death (9mo)	Negative
7	18	None	Patent (1y, 7mo)	Alive (2y)	Positive
8	50	Bile leakage	Patent (10mo)	Alive (2y, 7mo)	Positive

IVC, inferior vena cava; LHV, left hepatic vein; HV, hepatic vein.

## Discussion

We examined the cases of eight patients who underwent liver surgery combined with IVC or HV resection, followed by vessel reconstruction with artificial vascular grafts, and explored the validity of the use of ePTFE grafts in liver surgery. There were no surgical complications accompanying the use of artificial vascular grafts and all grafts in patients were patent. Therefore, liver resection combined with vessel reconstruction of the IVC or HV with subsequent reconstruction using ePTFE grafts can be performed safely in selected patients. Furthermore, our series contained two cases of over five-year survival (Table 2), although the untreated option for hepatic malignancy offers only a median survival of about three months [3] and chemotherapy does not offer a cure. Therefore, liver tumors invading the IVC or HV roots are not necessarily a contraindication for hepatectomy.

In liver surgery, there are a few cases that can only be cured by performing resection and reconstruction of the IVC or HV. Primary closure is impossible, and patch closure is often difficult during IVC reconstructions when the tumor invasion range is large; thus, reconstruction with an artificial vascular graft is often required [4]. Experience of living donor liver transplantation has shown that HV reconstruction using a vascular graft is sometimes necessary to reduce the residual liver congestion area and ensure effective liver volume [5]. Many graft materials can be used, including cryopreserved veins [6], the external iliac vein [7], the great saphenous vein [8], the ovarian vein [9], or an artificial vascular graft [10]. Autologous or cryopreserved veins work well in these grafts, if available, but they are often unavailable because there is a limit to the size and distance. Furthermore, surgical procedures for using these veins are often complex and time consuming. The artificial vascular graft has the advantages of freedom of length and diameter and can be used to respond to various situations. However, use of an artificial vascular graft has the potential risk of infection and thrombosis. Therefore, we evaluated our experience of using artificial vascular grafts in liver surgery.

In the cases presented here, complications occurred in four patients: two patients experienced bile leakage, one experienced a wound infection, and one experienced pleural effusion. However, there were no complications involving the ePTFE grafts. Graft infection is the most serious complication related to the use of ePTFE grafts. In our series, biliary reconstruction by Roux-en-Y hepaticojejunostomy was performed in one case and reoperations were performed for bile leakage on postoperative day 1 in two cases, but graft infection did not occurred in any of the patients. Arii *et al*. [4] reported 11 cases of hepatectomy with IVC resection, followed by IVC reconstruction with an ePTFE graft, with no graft infections. Azoulay *et al*. [11] reported 22 cases of hepatectomy with IVC resection and reconstruction, of which 10 cases were reconstructed with an ePTFE graft. They also reported no graft infections. Hemming *et al*. [12] reported 22 cases of hepatectomy with IVC resection and reconstruction, of which 16 cases were reconstructed with an ePTFE tube graft or an ePTFE patch (including five biliary reconstruction cases and two postoperative bile leak cases); graft infection did not occur in any of these cases, as with our study. These reports only describe IVC reconstruction, but we have also performed HV reconstructions with an ePTFE graft in liver resection in this report. Using an ePTFE graft in living donor liver transplantation has also been reported, and there has been no evidence of clinical infectious complications derived from the use of ePTFE grafts [13]. As seen in the cases reported here, the ePTFE graft can be used without serious infectious complications in most cases. The ePTFE graft is considered to have strong resistance to infection compared with other artificial vascular grafts, such as Dacron grafts [14,15]. However, there have been reports that ePTFE may be susceptible to infection [16]. One graft infection case that occurred during multiple organ failure resulting from a postoperative duodenal perforation has been reported [17]. Therefore, the use of ePTFE graft in the case of a contaminated operation must be considered carefully. In our series, two cases with postoperative bile leakage were treated one day after surgery. This prompt treatment may be important for the prevention of graft infection.

Another important issue with ePTFE grafts is graft patency. In our series, all the cases showed graft patency throughout the observation periods. In liver surgery using an ePTFE graft for IVC reconstruction, the ePTFE graft has been reported to show very high patency rates [4,11,12,18], which is consistent with our results. Recently, Matsuda *et al*. [19] reported combined resection of the IVC with replacement by an ePTFE graft in a living donor liver transplantation for hepatocellular carcinoma beyond the Milan criteria. They observed no complication related to the ePTFE graft. The ePTFE graft is considered to resist respiratory compression and graft collapse [18], resulting in few cases of graft thrombosis development. Another advantage of the ePTFE graft for IVC reconstruction is that it generally prevents narrowing of the lumen that could occur by compression of the graft during liver regeneration [15]. Therefore, many authors prefer to use the ePTFE graft for IVC reconstruction, rather than other graft materials. Good graft patency has also been reported when using an ePTFE graft for HV reconstruction combined with liver resection [10]. Use of the ePTFE graft for MHV reconstruction in living donor liver transplantation has also been reported [13]. In this report, the early patency rate of the ePTFE graft was good, whereas the late patency rate was reduced. However, the late obstructions of these ePTFE graft were all asymptomatic and had no impact on postoperative liver congestion, liver regeneration, or patient survival. With respect to the duration of graft patency, one to two weeks is considered to be enough to maintain adequate liver graft function because intrahepatic venous collateral can be expected to develop within one week of the operation [20]. Another report has described the conversion of the portal tract to an outflow channel within one hour, and intrahepatic venous collateral formation within two weeks when the hepatic vein was occluded [21]. Luminal thrombus formation is uncommon when an ePTFE graft is used for IVC reconstruction because the IVC can be classified as a high-flow vessel. By contrast, the MHV may be a low-flow vessel, resulting in the possibility of luminal thrombus formation [22]. Although a short period of graft patency may be acceptable in a case of HV reconstruction, warfarin should be used for long patency for cases of chronic hepatitis and liver cirrhosis, in order to secure liver volume. We did not stop warfarin because there are no established guidelines for the appropriate duration of the anticoagulant therapy.

The important feature of liver resection using an artificial vascular graft is to secure tumor-free margins for patients in whom tumor-free margins cannot be obtained with standard liver resection. Recently, Hemming *et al*. [23] reported that the only possible method for obtaining tumor-free margins would be the use of *ex vivo* resection techniques if there was complex involvement of the IVC, hepatic veins, and/or portal structures. *Ex vivo* resection is rarely needed because most tumors can be resected with less technically demanding techniques; however, *ex vivo* resection may be effective for patients in whom obtaining tumor-free margins during an *in situ* aggressive surgical procedure is not possible.

Hepatocellular carcinomas often expand with a capsule and form a tumor thrombus into intrahepatic vessels rather than directly invading the vessels. By contrast, adenocarcinomas, such as cholangiocarcinomas and metastatic tumors from colon cancer, tend to invade surrounding organs directly [24]. Because various situations can lead to a case of liver surgery requiring vascular resection, accurate preoperative assessment is essential. Recently, 3D-CT was reported to be useful for evaluating the positions of liver tumors and vessels precisely. It yields an accurate preoperative assessment and is a useful aid for surgical planning [25-27]. We also take advantage of 3D-CT in preoperative simulations to avoid unnecessary vascular resections or to prepare a vascular graft before surgery. With respect to the IVC invasion, histological invasion of the replaced IVC was confirmed in four cases in our series, and the accuracy rate for preoperative diagnosis of IVC invasion was 66.7% (four out of six). Although 3D-CT is generally considered to be useful for preoperative simulation [25], novel diagnostic procedures for vascular invasion are expected.

## Conclusions

Our results indicate liver resection combined with IVC or HV vessel reconstruction using an ePTFE graft can be performed relatively safely in selected patients. Furthermore, precise preoperative assessment and surgical planning are mandatory to safely perform liver surgeries requiring venous reconstruction.

## Abbreviations

3D-CT: three-dimensional computed tomography; CT: computed tomography; ePTFE: expanded polytetrafluoroethylene; HV: hepatic vein; ICGR15: indocyanine green retention rate at 15 min; IVC: inferior vena cava; LHV: left hepatic vein; MHV: middle hepatic vein.

## Competing interests

The authors declare that they have no competing interests.

## Authors’ contributions

TO and TK designed the research. TO, TK, HY, TK, KW, YT, HK, and AT contributed to acquisition of data. TO, TK, and AT analyzed and interpreted data. All authors read and approved the final manuscript.
